# Objective and subjective cognitive outcomes one year after COVID‐19

**DOI:** 10.1002/acn3.52149

**Published:** 2024-07-19

**Authors:** Laura Zamarian, Verena Rass, Elisabeth Goettfried, Valentina Mayr, Federico Carbone, Philipp Kindl, Margarete Delazer, Atbin Djamshidian, Alessandra Fanciulli, Philipp Mahlknecht, Beatrice Heim, Marina Peball, Alois Josef Schiefecker, Klaus Seppi, Judith Löffler‐Ragg, Ronny Beer, Bettina Pfausler, Stefan Kiechl, Raimund Helbok

**Affiliations:** ^1^ Department of Neurology Medical University of Innsbruck Innsbruck Austria; ^2^ Department of Neurology Provincial Hospital of Kufstein Kufstein Austria; ^3^ Department of Internal Medicine II Medical University of Innsbruck Innsbruck Austria; ^4^ Department of Pneumology State Hospital of Hochzirl–Natters Natters Austria; ^5^ Department of Neurology Johannes Kepler University Linz Linz Austria

## Abstract

**Objective:**

This study aimed to evaluate subjective cognitive, physical, and mental health symptoms as well as objective cognitive deficits in COVID‐19 patients 1 year after infection.

**Methods:**

This was a cross‐sectional study. Seventy‐four patients, who contracted a SARS‐CoV‐2 infection in 2020, underwent an in‐person neuropsychological assessment in 2021. This included standardized tests of memory, attention, and executive functions. In addition, participants also responded to scales on subjective attention deficits, mental health symptoms, and fatigue. Patients' scores were compared to published norms.

**Results:**

Patients (*N* = 74) had a median age of 56 years (42% female). According to the initial disease severity, they were classified as mild (outpatients, 32%), moderate (hospitalized, non‐ICU‐admitted, 45%), or severe (ICU‐admitted, 23%). Hospitalized patients were more often affected than outpatients. In general, deficits were most common in attention (23%), followed by memory (15%) and executive functions (3%). Patients reported increased levels of fatigue (51%), anxiety (30%), distractibility in everyday situations (20%), and depression (15%). An additional analysis suggested an association between lower scores in an attention task and hyperferritinemia. As indicated by a hierarchical regression analysis, subjective distractibility was significantly predicted by current anxiety and fatigue symptoms but not by objective attention performance (final model, adj‐*R* ^2^ = 0.588, *P* < 0.001).

**Interpretation:**

One year after infection, COVID‐19 patients can have frequent attention deficits and can complain about symptoms such as fatigue, anxiety, and distractibility. Anxiety and fatigue, more than objective cognitive deficits, have an impact on the patients' experienced impairments in everyday life.

## Introduction

Severe acute respiratory syndrome coronavirus 2 (SARS‐CoV‐2) infection, also known as coronavirus disease 2019 (COVID‐19), has been found to be associated with symptoms persisting even beyond 4–12 weeks after disease onset.^1^ Apart from pulmonary and other organic symptoms, post‐COVID‐19 condition may include neurological, cognitive, and neuropsychiatric symptoms such as sleep disturbances, headache, anosmia, “brain fog”, fatigue, anxiety, and depression.^2^, ^3^, ^4^, ^5^, ^6^, ^7^ It has been argued that these post‐COVID‐19 symptoms may be a consequence of a persistent pro‐inflammatory response, hypoxic–ischemic injury, or changes in neurotransmitters.^8^ As subjective cognitive deficits may persist well beyond the physical and functional recovery, systematic assessments of post‐COVID‐19 cognitive symptoms in the long term are warranted. Studies using comprehensive neuropsychological assessments revealed reduced attention and processing speed 1 year after disease onset.^9^ In an eye‐tracking study, we showed that at 1‐year follow‐up COVID‐19 patients who required hospitalization in the acute phase performed worse than healthy controls and patients who were managed as outpatients in tasks assessing response inhibition, attention, and working memory.^10^

In this study, we aimed to quantify neurocognitive deficits in COVID‐19 patients 1 year after infection during the first wave of the pandemic by using a comprehensive neuropsychological test battery. Co‐variates included subjective cognitive performance, fatigue, mental health symptoms, and associated systemic inflammatory responses as expressed by serum ferritin levels. Patients' performances were compared to published norms. We hypothesized that cognitive deficits are more frequent in hospitalized patients than in non‐hospitalized patients. Since results of previous studies suggest an improvement of objective cognitive deficits over time,^11^ while subjective physical, mental, and cognitive symptoms are prevalent and persisting in COVID‐19 patients in the long term,^12^, ^13^ we hypothesized a discrepancy between subjective and objective cognitive deficits. We also hypothesized that subjective cognitive deficits are possibly influenced by current anxiety, depression, and fatigue symptoms.

## Methods

### Participants

This was a cross‐sectional study. One year after SARS‐CoV‐2 infection, 74 patients underwent an in‐person neuropsychological background assessment between April and July 2021. A subgroup of these patients also participated in previous studies.^10^, ^13^ Study inclusion criteria were as follows: (1) a SARS‐CoV‐2 infection between March and June 2020, confirmed with PCR testing; (2) hospitalization or patient management with symptoms persisting for at least 6 weeks after disease onset; (3) age greater than 18 years; and (4) fluent German language. At the time of acute SARS‐CoV‐2 infection, patients were not vaccinated and had the wild type or alpha variant. The study protocol was approved by the local ethics committee (Medical University of Innsbruck, EK‐no. 1103/2020) and registered on ClinicalTrials.gov (NCT05025839). Participants provided written informed consent according to the declaration of Helsinki.

### Study procedure and materials

The neuropsychological assessment included objective cognitive measures and scales assessing self‐perceived cognitive, physical, and mental health symptoms.

#### Objective cognitive outcomes

The sum score in the Montreal Cognitive Assessment (MoCA)^14^ was taken as measure of global cognitive status. We assessed alertness and divided attention through the computerized Tests of Attentional Performance battery (TAP; https://www.psytest.de). The Digit Span Forward and the Digit Span Backward subtests of the Wechsler Memory Scale^15^ were used as measures of verbal attention span and verbal working memory. Psychomotor speed and cognitive flexibility were tested through the Trail Making Test Part A (TMT‐A) and Part B (TMT‐B).^16^ Semantic verbal fluency (animals/min) and phonemic verbal fluency (s‐words/min) were assessed through the Regensburger Wortflüssigkeitstest (RWT).^17^ Verbal memory performance was evaluated through the Neuropsychological Assessment Battery (NAB).^18^

We defined MoCA scores lower than 26/30 as suggestive of cognitive impairments.^14^ We compared the individual's performances in other cognitive measure to published age‐stratified (and in the case of TMT, education‐stratified) norms. In line with Lezak,^19^ we then classified performance as slightly‐to‐severely impaired if laying below the 10th percentile of norms. We also carried out an analysis at the domain level and looked at cases where scores below the 10th percentile of norms were found in half or more of the measures used to assess a specific cognitive domain.

#### Subjective cognitive, physical, and mental health outcomes

The Hospital Anxiety and Depression Scale (HADS)^20^ measures subjective levels of anxiety and depression symptoms during the last week. Scores in each 7‐item subscale range from 0 to 21. The Fatigue Assessment Scale (FAS)^21^ is a 10‐item questionnaire assessing mental health and physical fatigue. The sum score ranges from 10 to 50. The FEDA (Fragebogen Erlebter Defizite der Aufmerksamkeit)^22^ assesses subjective attention deficits in everyday situations and comprises three subscales (tot. 27 items): one on distractibility and retardation in mental tasks (FEDA‐1; e.g., watching movies), one on tiredness and retardation in practical activities (FEDA‐2; e.g., doing the laundry), and one on reduction of drive (FEDA‐3; e.g., interest in hobbies). Scores range from one to 65 in the first subscale, from one to 40 in the second subscale, and from one to 30 in the third subscale, with lower scores indicating lower levels of functioning.

Scores in each scale of the HADS^20^ equal to or higher than eight were considered as suggestive of mild to clinically meaningful anxiety or depression disorders. A score in the FAS higher than 21 was taken as indicative of a clinically relevant fatigue.^21^ We considered scores ≤40 in FEDA‐1, ≤27 in FEDA‐2, and ≤17 in FEDA‐3, which lay below the 10th percentile of norms,^22^ as indicative of slight‐to‐severe subjective cognitive deficits in everyday situations.

### Statistical analysis

If not differently specified, statistical analyses were conducted with SPSS (IBM Statistics, Version 27.0). Statistical significance was set at α = 0.05. We give categorical variables in counts and percentages, and continuous variables as medians and interquartile ranges (IQRs). Frequency distributions were assessed by binomial or the chi‐square test (Yates' correction; http://www.quantpsy.org/chisq/chisq). Linear variables were compared between groups or conditions through non‐parametric tests (Wilcoxon test, Kruskal–Wallis test, Mann–Whitney test). Missing data were excluded from analysis and indicated appropriately. Results are given for different disease severity groups defined according to the required treatment setting during the initial disease course: 1) “mild” (outpatients who sought medical help), “moderate” (admitted to the normal ward), and “severe” (admitted to the intensive care unit, ICU). In a subgroup analysis, results on objective cognitive measures are also given for patients with hyperferritinemia and patients with normal serum ferritin levels at 1‐year follow‐up. As we were interested in a possible influence of objective attention deficits on distractibility in everyday situations, we performed a hierarchical regression analysis on the whole sample with the FEDA‐1 score as dependent variable. Objective attention measures (verbal attention span, reaction times [RTs] in intrinsic alertness, and omissions in divided attention) were entered as predictors in Block 1. Ratings in the HADS were entered in Block 2 to control for the influence of anxiety and depression symptoms. Ratings in the FAS were entered in Block 3 to control for the effects of fatigue. We focused on the FEDA‐1 subscale (distractibility) as items of the FEDA‐2 (tiredness) and FEDA‐3 (drive reduction) subscales refer to difficulties in everyday situations that partially overlap with fatigue and depression symptoms as measured with the FAS and HADS. Also, in Block 1 we did not enter phasic alertness scores as predictor, since this measure demonstrated a high correlation with the intrinsic alertness scores during verification of potential collinearity.

## Results

Demographics and clinical characteristics are given in Table 1. Seventy‐four patients completed the neuropsychological assessment 1 year after infection (median 420 days, IQR: 407–438). At the time of this investigation, patients showed a good functional outcome (median score in the Glasgow Outcome Scale‐Extended: 8; median score in the modified Rankin Scale: 1). The median age was 56 years, and the median education was 13 years. Thirty‐one patients (42%) were female. According to the initial disease severity, 24 patients (32%) were managed as outpatients (“mild”), 33 patients (45%) required admission to the normal ward (“moderate”), and 17 patients (23%) required ICU admission (“severe”). There were significant differences across disease severity grades in age, education, and sex distribution (for details, see Table 1). Hospitalized patients were older and had a lower education. The frequency of female patients was higher in the hospitalized group than in the outpatient group. Age, education, and sex distribution were comparable between ICU‐admitted patients and non‐ICU‐admitted patients.

**Table 1:** Demographics and clinical characteristics according to initial COVID‐19 severity grade as well as for the whole sample.

	All *N* = 74	Mild *n* = 24 (32%)	Moderate *n* = 33 (45%)	Severe *n* = 17 (23%)	Group effect^1^, ^2^ *P*‐value	Comparison 1 versus 2^1^, ^3^ *P*‐value	Comparison 1 versus 3^1^, ^3^ *P*‐value	Comparison 2 versus 3^1^, ^3^ *P*‐value
Characteristics								
Median age, years (IQR)	56 (48–65)	48 (38–55)	64 (55–73)	55 (50–65)	<0.001	<0.001	0.004	0.078
Median education, years (IQR)	13 (11–15)	14 (12–16)	12 (11–15)	12 (11–13)	0.039	0.023	0.033	0.934
Female sex	31 (42%)	17 (71%)	10 (30%)	4 (24%)	<0.001	<0.001	<0.001	0.426
Median BMI at baseline (IQR)	26 (24–29)	25 (23–29)	27 (26–30)	26 (24–29)	0.233	–	–	–
Premedical history								
Cardiovascular disease	26 (35%)	1 (4%)	16 (48%)	9 (53%)	<0.001	<0.001	<0.001	0.572
Arterial hypertension	20 (27%)	1 (4%)	13 (39%)	6 (35%)	<0.001	<0.001	<0.001	0.660
Pulmonary disease	14 (19%)	5 (21%)	6 (18%)	3 (18%)	0.914	–	–	–
Endocrinological disease	30 (40%)	5 (21%)	18 (54%)	7 (41%)	<0.001	<0.001	0.004	0.089
Hypercholesterolemia	16 (22%)	1 (4%)	12 (36%)	3 (18%)	<0.001	<0.001	0.003	0.007
Diabetes mellitus II	12 (16%)	1 (4%)	8 (24%)	33 (18%)	0.001	<0.001	0.003	0.386
Chronic kidney disease	4 (5%)	0 (0%)	2 (6%)	2 (12%)	0.004	0.038	0.001	0.217
Chronic liver disease	4 (5%)	0 (0%)	2 (6%)	2 (12%)	0.004	0.038	0.001	0.217
Malignancy	8 (11%)	1 (4%)	6 (18%)	1 (6%)	0.003	0.003	0.746	0.017
Immunodeficiency	2 (3%)	1 (4%)	0 (0%)	1 (6%)	0.939	–	–	–
Pre‐existing neurological diseases^4^	18 (24%)	5 (21%)	9 (27%)	4 (24%)	0.699	–	–	–
Treatment and hospital course								
Oxygen	33 (42%)^5^	0 (0%)^6^	17 (52%)	16 (100%)^6^				
Mechanical ventilation	15 (20%)^6^	0 (0%)	0 (0%)	15 (94%)^6^				
Steroids	12 (17%)^5^	1 (4%)^6^	6 (18%)	5 (31%)^6^				
Median hospital stay, days (IQR)	–	–	10 (6–13)	30 (19–42)				
Median ICU days (IQR)	–	–	–	16 (10–24)				
Functional outcome at 1‐year follow‐up								
Median GOS‐E (IQR)	8 (7–8)^7^	8 (7–8)^6^	8 (7–8)^5^	7 (7–8)^5^	0.352	–	–	–
Median mRS (IQR)	1 (0–1)^7^	0 (0–1)^6^	0 (0–1)^5^	1 (0–1)^5^	0.571	–	–	–
Hyperferritinemia at 1‐year follow‐up^8^	12 (21%)	1 (2%)	9 (16%)	2 (4%)	0.001	0.001	0.010	0.678

Mild, outpatients; Moderate, hospitalized patients, who were not admitted to the intensive care unit (ICU); Severe, ICU‐admitted patients; IQR, interquartile range; MoCA, Montreal Cognitive Assessment; GOS‐E, Glasgow Outcome Scale‐Extended; mRS, modified Rankin Scale; (−), not applicable.

^1^ Sex distributions were compared by means of the chi‐square test (Yates' correction).

^2^ The main effect of group was analyzed through Kruskal–Wallis test.

^3^ Pairwise group comparisons in variables other than sex distribution were conducted through Mann–Whitney test.

^4^ None of these patients had a pre‐existing diagnosis of mild cognitive impairment or dementia; none had a neuropsychological assessment prior to COVID‐19.

^5^ Two missing values.

^6^ One missing value.

^7^ Five missing values.

^8^ Serum ferritin levels were determine in 56/74 patients, and hyperferritinemia was defined as serum ferritin levels above 400 *μ*g/L.

### Objective cognitive outcomes

Table 2 reports counts and percentages of deficits in each single cognitive measure. Medians and IQRs of raw scores are reported as Tables S1 and S2. None of the outpatients but around 30% of patients in each hospitalized group showed deficits in the MoCA. Significant group differences emerged in verbal attention span, intrinsic alertness, divided attention, verbal working memory, phonemic fluency, and verbal memory but not in phasic alertness, semantic fluency, psychomotor speed, and cognitive flexibility. In general, cognitive deficits were more frequent in hospitalized patients than in outpatients. There were very few differences between ICU‐admitted patients and non‐ICU‐admitted patients (deficits in delayed free recall: non‐ICU‐admitted > ICU‐admitted, *P* = 0.043; deficits in verbal attention span: ICU‐admitted > non‐ICU‐admitted, *P* = 0.008).

**Table 2:** Frequency of scores in each objective cognitive measure below the 10th percentile of age‐scaled/education‐scaled norms.

	All *N* = 74	Mild *n* = 24 (32%)	Moderate *n* = 33 (45%)	Severe *n* = 17 (23%)	Group effect^1^ *P*‐value	Comparison 1 versus 2^1^ *P*‐value	Comparison 1 versus 3^1^ *P*‐value	Comparison 2 versus 3^1^ *P*‐value
MoCA	15 (20%)	0 (0%)	10 (30%)	5 (29%)	<0.001	<0.001	<0.001	1
Attention								
Verbal attention span, digit span forward (WMS)	8 (11%)	1 (4%)	3 (9%)	4 (24%)	<0.001	0.251	<0.001	0.008
Intrinsic alertness, median RTs (TAP)	17 (23%)	3 (12%)	9 (27%)	5 (29%)	0.013	0.012	0.005	0.874
Phasic alertness, median RTs (TAP)	22 (30%)	7 (29%)	9 (27%)	6 (35%)	0.540	–	–	–
Divided attention, omissions (TAP)	11 (15%)^2^	2 (8%)	7 (21%)^2^	2 (12%)	0.042	0.016	0.480	0.127
Executive functions								
Verbal working memory, digit span backward (WMS)	5 (7%)	0 (0%)	3 (9%)	2 (12%)	0.007	0.006	0.001	0.644
Semantic verbal fluency, animals/min (RWT)	1 (1%)^3^	1 (4%)^3^	0 (0%)	0 (0%)	0.099	–	–	–
Phonemic verbal fluency, s‐words/min (RWT)	5 (7%)^3^	3 (13%)^3^	1 (3%)	1 (6%)	0.045	0.019	0.148	0.495
Psychomotor speed (TMT‐A)	2 (3%)	0 (0%)	1 (3%)	1 (6%)	0.112	–	–	–
Cognitive flexibility (TMT‐B)	5 (7%)^3^	1 (4%)	3 (9%)^3^	1 (6%)	0.506	–	–	–
Memory								
Verbal learning (NAB)	12 (16%)	1 (4%)	7 (21%)	4 (24%)	<0.001	<0.001	<0.001	0.734
Verbal immediate free recall (NAB)	13 (18%)	3 (12%)	7 (21%)	3 (18%)	0.313	–	–	–
Verbal delayed free recall (NAB)	12 (16%)	2 (8%)	8 (24%)	2 (12%)	0.008	0.004	0.480	0.043
Verbal correct recognition (NAB)	9 (12%)	1 (4%)	5 (15%)	3 (18%)	0.014	0.016	0.003	0.703

Mild, outpatients; Moderate, hospitalized patients, who were not admitted to the intensive care unit (ICU); Severe, ICU‐admitted patients; MoCA, Montreal Cognitive Assessment; WMS, Wechsler Memory Scale; TAP, Tests of Attentional Performance; RWT, Regensburger Word fluency Test; TMT, Trail Making Test; NAB, Neuropsychological Assessment Battery; RTs, reaction times; (−), not applicable.

^1^ Frequency distributions were compared by means of the chi‐square test (Yates' correction).

^2^ Two missing values.

^3^ One missing value.

**Figure 1:**
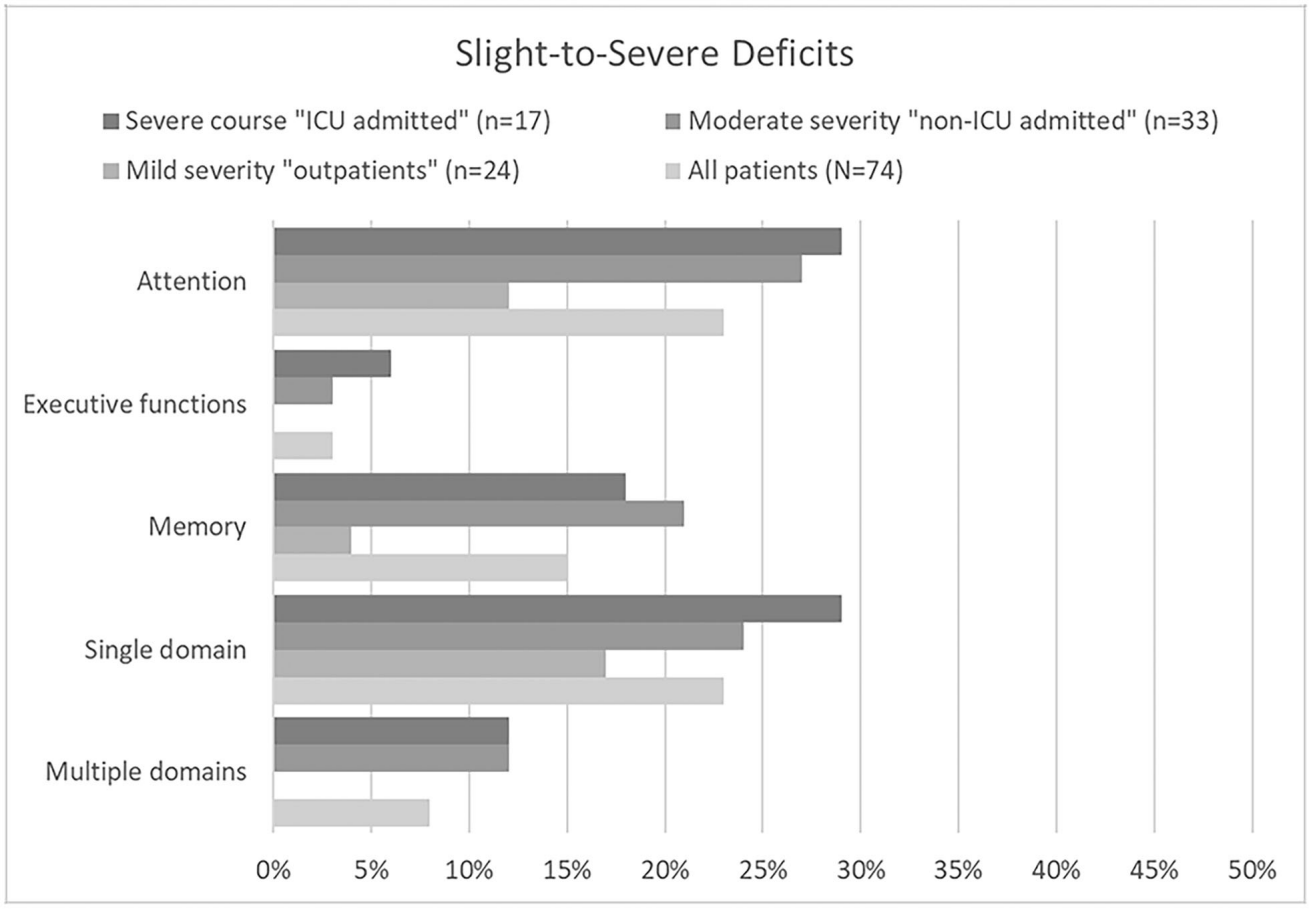
Frequency of slight‐to‐severe objective cognitive deficits at the domain level. ICU, intensive care unit.

Figure 1 reports percentages of impairments at the domain level. Exact counts and *P*‐values are given in Table 3. Attention deficits were most prevalent, followed by deficits in memory and executive functions. Overall, 23% of patients had deficits in a single domain; in 8%, multiple domains were affected. Deficits in a single domain were equally frequent across disease severity grades. None of the outpatients but 12% of the hospitalized patients showed deficits in multiple domains.

**Table 3:** Frequency of slight‐to‐severe objective cognitive deficits at the domain level.

	All *N* = 74	Mild *n* = 24 (32%)	Moderate *n* = 33 (45%)	Severe *n* = 17 (23%)	Group effect^1^ *P*‐value	Comparison 1 versus 2^1^ *P*‐value	Comparison 1 versus 3^1^ *P*‐value	Comparison 2 versus 3^1^ *P*‐value
Attention	17 (23%)	3 (12%)	9 (27%)	5 (29%)	0.013	0.012	0.005	0.874
Executive functions	2 (3%)	0 (0%)	1 (3%)	1 (6%)	0.112	–	–	–
Memory	11 (15%)	1 (4%)	7 (21%)	3 (18%)	0.003	<0.001	0.003	0.722
Single domain	17 (23%)	4 (17%)	8 (24%)	5 (29%)	0.183	–	–	–
Multiple domains	6 (8%)	0 (0%)	4 (12%)	2 (12%)	0.004	0.001	0.001	0.828

Deficits at the domain level refer to cases where scores were below the 10th percentile of age‐scaled/education‐scaled norms in half or more of the measures used to assess a specific cognitive domain. Mild, outpatients; Moderate, hospitalized patients, who were not admitted to the intensive care unit (ICU); Severe, ICU‐admitted patients; (−), not applicable.

^1^ Frequency distributions were compared by means of the chi‐square test (Yates' correction).

In general, deficits were more common in hospitalized patients than in outpatients, while there were no differences between ICU‐admitted patients and non‐ICU‐admitted patients.

To strengthen the results of the comparison of the individual's performances to published norms, between March 2021 and November 2022, we recruited 30 age‐matched healthy controls who did not have allegedly yet contracted the SARS‐CoV‐2 infection (as per history taking). Data and results are reported as Tables S1 and S2. In comparison with controls, COVID‐19 patients showed lower scores in the MoCA and in several measures of attention (verbal attention span, intrinsic alertness, and divided attention, but not phasic alertness), executive functions (verbal working memory, verbal fluency, and cognitive flexibility, but not psychomotor speed), and memory (learning, free recall, and recognition).

### Subjective cognitive, physical, and mental health outcomes

Table 4 reports counts and percentages of ratings deviating from cutoff scores that are suggestive of increased levels of subjective symptoms. Medians and IQRs of raw scores are reported as Tables S1 and S2. Increased levels of fatigue, anxiety, and depression were reported in 51%, 30%, and 15% of the patients, respectively. Increased subjective distractibility, tiredness, and drive reduction in everyday situations were reported in 20%, 16%, and 10% of the cases, respectively.

**Table 4:** Frequency of increased levels of subjective cognitive, physical, and mental health symptoms.

	All *N* = 74	Mild *n* = 24 (32%)	Moderate *n* = 33 (45%)	Severe *n* = 17 (23%)	Group effect^1^ *P*‐value	Comparison 1 versus 2^1^ *P*‐value	Comparison 1 versus 3^1^ *P*‐value	Comparison 2 versus 3^1^ *P*‐value
Self‐perceived cognitive symptoms								
Subjective distractibility (FEDA‐1)	15 (20%)^2^	6 (26%)^2^	4 (12%)	5 (29%)	0.015	0.019	0.874	0.008
Subjective tiredness (FEDA‐2)	12 (16%)^2^	2 (9%)^2^	5 (15%)	5 (29%)	0.002	0.276	<0.001	0.026
Subjective drive reduction (FEDA‐3)	7 (10%)^2^	3 (13%)^2^	3 (9%)	1 (6%)	0.343	–	–	–
Self‐perceived mental health symptoms								
Anxiety (HADS)	22 (30%)	6 (25%)	9 (27%)	7 (41%)	0.045	0.872	0.024	0.052
Depression (HADS)	11 (15%)	4 (18%)	2 (6%)	5 (29%)	<0.001	0.027	0.064	<0.001
Self‐perceived physical symptoms								
Fatigue (FAS)	37 (51%)^2^	10 (42%)	16 (47%)^2^	11 (65%)	0.005	0.569	0.002	0.015

Increased levels of subjective cognitive, physical, and mental health symptoms are defined as scores in validated scales deviating from published cutoff scores. Mild, outpatients; Moderate, hospitalized patients, who were not admitted to the intensive care unit (ICU); Severe, ICU‐admitted patients; ICU, intensive care unit; HADS, Hospital Anxiety and Depression Scale – German version; FEDA, Fragebogen Erlebter Defizite der Aufmerksamkeit; FAS, Fatigue Assessment Scale; (−), not applicable.

^1^ Frequency distributions were compared by means of the chi‐square test (Yates' correction).

^2^ One missing value.

Increased levels of tiredness, fatigue, and anxiety were more frequently recorded in ICU‐admitted patients than in other patients. Increased levels of depression and distractibility were more common in outpatients and ICU‐admitted patients than in non‐ICU‐admitted patients. Pronounced drive reduction was reported equally frequent across disease severity grades.

### Association between objective cognitive outcomes and hyperferritinemia

Results of a subgroup analysis (*n* = 56) indicated that patients with hyperferritinemia at the 1‐year follow‐up performed worse than patients with normal ferritin levels in a verbal attention span task (see Table 5), although performance of only one patient with hyperferritinemia was laying below the 10th percentile of norms. The majority of patients with hyperferritinemia had a moderate initial disease course (9/12, 75%). Patients with hyperferritinemia at 1‐year follow‐up had elevated ferritin levels at previous examinations as well (6 weeks: 11/12, 92%; 3 months: 9/12, 75%; 6 months: 7/12, 58%).

**Table 5:** Median scores and interquartile ranges (IQRs) in objective cognitive measures and self‐rated scales for patients with normal ferritin levels and patients with hyperferritinemia at 1‐year follow‐up.

	Normal ferritin levels (*n* = 44)	Hyperferritinemia (*n* = 12)	*P*‐value^1^	Cohen's *d*
MoCA	27 (27–29)	26 (24–28)	0.162	0.27
Attention				
Verbal attention span, digit span forward (WMS)	7 (6–9)	6 (5–6)	0.004	0.92
Intrinsic alertness, median RTs in msec (TAP)	267 (230–322)	255 (227–301)	0.625	0.14
Phasic alertness, median RTs in msec (TAP)	265 (234–299)	248 (228–284)	0.369	0.20
Divided attention, omissions (TAP)^2^	1 (0–1)	1 (0–2)	0.227	−0.22
Executive functions				
Verbal working memory, digit span backward (WMS)	6 (5–8)	6 (4–6)	0.121	0.56
Semantic verbal fluency, animals/min (RWT)^3^	25 (22–31)	21 (18–28)	0.077	0.62
Phonemic verbal fluency, s‐words/min (RWT)^3^	13 (9–17)	11 (9–14)	0.208	0.46
Psychomotor speed, RTs in sec (TMT‐A)^3^	26 (22–34)	26 (24–35)	0.975	0.14
Cognitive flexibility, RTs in sec (TMT‐B)^4^	65 (51–81)	64 (54–92)	0.629	−0.10
Memory				
Verbal learning (NAB)	22 (18–25)	21 (16–22)	0.298	0.37
Verbal immediate free recall (NAB)	7 (4–9)	6 (4–8)	0.651	0.17
Verbal delayed free recall (NAB)	7 (5–9)	5 (4–8)	0.546	0.17
Verbal correct recognition (NAB)	8 (6–10)	8 (4–10)	0.615	0.16
Subjective cognitive, physical, and mental health symptoms				
Subjective distractibility (FEDA‐1)^3^	52 (46–58)	56 (46–58)	0.984	−0.06
Subjective tiredness (FEDA‐2)^3^	36 (31–40)	35 (30–38)	0.545	0.17
Subjective drive reduction (FEDA‐3)^3^	26 (20–30)	24 (23–26)	0.479	0.14
Anxiety (HADS)	4 (2–8)	6 (2–8)	0.623	−0.09
Depression (HADS)	1 (1–4)	3 (1–5)	0.490	0.00
Fatigue (FAS)^3^	20 (15–24)	21 (19–28)	0.414	−0.18

Objective cognitive deficits refer to scores in objective cognitive measures that lay below the 10th percentile of age‐scaled/education‐scaled norms. Increased levels of subjective cognitive, physical, and mental health symptoms are defined as scores in validated scales deviating from published cutoff scores. Hyperferritinemia was defined as serum ferritin levels above 400 μg/L. MoCA, Montreal Cognitive Assessment; WMS, Wechsler Memory Scale; TAP, Tests of Attentional Performance; RWT, Regensburger Word fluency Test; TMT, Trail Making Test; NAB, Neuropsychological Assessment Battery; RTs, reaction times; HADS, Hospital Anxiety and Depression Scale – German version; FEDA, Fragebogen Erlebter Defizite der Aufmerksamkeit; FAS, Fatigue Assessment Scale.

^1^ Group comparisons were conducted through Mann–Whitney test.

^2^ Two missing values.

^3^ One missing value.

^4^ Three missing values.

### Factors associated with subjective distractibility in everyday life

Table 6 reports the coefficients of a hierarchical regression analysis where scores in FEDA‐1 (distractibility) were entered as dependent variable. Model 1 with scores in objective attention measures as predictors was significant and explained 11% of the variance in the FEDA‐1 scores (adj‐*R* ^2^ = 0.110; *F*(3,66) = 3.85, *P* = 0.013). Model 2 where scores in anxiety and depression scales were added to Model 1 explained an additional 41% of variance. This change in *R* ^2^ was significant (*F*(2,64) = 29.74, *P* < 0.001). In Model 2, anxiety emerged as the only significant predictor. Model 3 where scores in the fatigue scale were added to Model 2 explained an additional 7% of variance. This change in *R* ^2^ was also significant (*F*(1,63) = 10.94, *P* = 0.002). Anxiety and fatigue emerged as significant predictors in Model 3. In sum, 59% of variance in subjective attention deficits was explained by the final model (adj‐*R* ^2^ = 0.588; *F*(6,63) = 17.44, *P* < 0.001). Please notice that results do not change relevantly when controlling for age, education, and sex (results not shown).

**Table 6:** Coefficients of a hierarchical regression analysis where scores in FEDA‐1 (distractibility) are the dependent variable.

	Model	Non‐standardized coefficients		Standardized coefficients	*T*	*P*‐value	Collinearity	
		*B*	SE	*β*			Tollerance	VIF
1	(Costant)	55.245	6.637		8.324	0.000		
	Verbal attention span	0.413	0.724	0.067	0.570	0.571	0.945	1.058
	Intrinsic alertness (RTs)	−0.025	0.013	−0.244	−1.882	0.064	0.768	1.302
	Divided attention (omissions)	−0.615	0.449	−0.180	−1.370	0.175	0.744	1.344
2	(Costant)	63.755	4.981		12.800	0.000		
	Verbal attention span	−0.028	0.537	−0.004	−0.052	0.959	0.918	1.089
	Intrinsic alertness (RTs)	−0.016	0.010	−0.162	−1.690	0.096	0.751	1.331
	Divided attention (omissions)	−0.076	0.338	−0.022	−0.224	0.823	0.703	1.422
	Anxiety	−1.321	0.390	−0.485	−3.386	0.001	0.336	2.980
	Depression	−0.637	0.422	−0.227	−1.510	0.136	0.305	3.275
3	(Costant)	70.292	5.038		13.953	0.000		
	Verbal attention span	0.155	0.503	0.025	0.307	0.760	0.907	1.102
	Intrinsic alertness (RTs)	−0.010	0.009	−0.098	−1.071	0.288	0.717	1.395
	Divided attention (omissions)	−0.082	0.314	−0.024	−0.262	0.794	0.703	1.422
	Anxiety	−0.807	0.395	−0.297	−2.044	0.045	0.284	3.526
	Depression	−0.297	0.406	−0.106	−0.731	0.4607	0.286	3.500
	Fatigue	−0.602	0.182	−0.404	−3.308	0.002	0.400	2.501

## Discussion

In this cross‐sectional study, we assessed the frequency of objective cognitive deficits and of increased levels of subjective cognitive, physical, and mental health symptoms in 74 recovered COVID‐19 patients. The assessment took place 1 year after the patients contracted the SARS‐CoV‐2 infection in 2020. The main finding was that slight‐to‐severe deficits in attention, memory, and executive functions were evident in 23%, 15%, and 3% of the patients, respectively. Deficits in one or more cognitive domains were more common in hospitalized patients than in outpatients (outpatients: 17%; non‐ICU‐admitted: 36%; ICU‐admitted: 42%). Increased levels of subjective fatigue, anxiety, and depression were reported in 51%, 30%, and 15% of the patients, respectively. Increased distractibility, tiredness, and drive reduction in everyday situations were reported in 20%, 16%, and 10% of the patients, respectively.

As COVID‐19 patients often report about experiencing cognitive changes weeks to months after the SARS‐CoV‐2 infection, we were interested into whether subjective distractibility in everyday situations was related to objective attention performance. Results of a hierarchical regression analysis showed that performance in objective attention measures explained only 11% of variance in ratings of subjective attention deficits. None of the objective attention measures emerged as significant predictor, when ratings of self‐perceived fatigue, anxiety, and depression symptoms were entered into the regression model. Mental health symptoms could explain 41% of variance, fatigue an additional 7%. The final model explained 59% of variance in ratings of subjective attention deficits, with anxiety and fatigue emerging as significant predictors.

Evidence from the COVID‐19 pandemic as well as from previous pandemics and epidemics^5^, ^23^, ^24^ points to long‐term persistence of neurological sequelae, among which cognitive dysfunction. Following a recent systematic review and meta‐analysis,^5^ there is a close association between cognitive deficits and SARS‐CoV‐2 infection. COVID‐19 patients with no prior history of cognitive impairment show poorer general cognitive functioning, as measured with the MoCA, relative to controls without COVID‐19 up to 7 months after infection.^5^ Studies using more extensive neurocognitive assessments find impairments in particular in executive functions, attention, and memory up to 3 months after infection.^5^ Our study adds to these findings by showing that cognitive disorders may be detected even 1 year after SARS‐CoV‐2 infection with the wild type or alpha variant, and that they may affect to a different extent attention, memory, and executive functions, with attention deficits being the most frequent. In this study, results of the comparison of the individual's performances to published norms were strengthened by a group comparison between COVID‐19 patients and age‐matched controls without COVID‐19, which pointed to lower scores for the patient group in the majority of the objective cognitive measures used. Regarding the long‐term effects of the initial disease severity grade on cognition, some studies found an association between cognitive dysfunction and severe disease course, while others did not.^5^ Findings of this study suggest a higher frequency of objective cognitive deficits in hospitalized patients than in outpatients. However, since hospitalized patients were older than outpatients, we may not exclude age as an additional factor for cognitive impairment. Moreover, multiple risk factors may have contributed to the cognitive outcome in ICU‐admitted patients, including disease severity, pre‐existing comorbidities, intervention (e.g., prolonged sedation, corticosteroids, and mechanical ventilation), and complications such as renal or hepatic failure. As indicated by a study of Mayerhöfer et al.,^25^ which included the ICU patients assessed in our study, renal and hepatic failures are a frequent ICU complication affecting up to 12% and 8% of the patients, respectively. Longitudinal studies demonstrate that impairments in physical, cognitive, and/or mental health status may persist in the long term (at least 1 to 5 years) after hospitalization in non‐COVID‐19 postintensive care survivors as well, clearly affecting their quality of life and subsequent healthcare management.^26^, ^27^

COVID‐19 patients frequently report experiencing in everyday situations cognitive difficulties, fatigue, anxiety, and depression symptoms even months apart from the infection.^11^, ^28^, ^29^, ^30^ For example, a longitudinal prospective multicenter cohort study found persistent complaints of fatigue (41%), cognitive impairment (28%), anxiety (19%), and depression symptoms (15%) in hospitalized (moderate to severe) COVID‐19 patients at 1‐year follow‐up.^28^ In a large longitudinal cohort study on 1276 hospitalized COVID‐19 survivors,^29^ the majority of patients had a good physical and functional recovery and had returned to the original work at 1‐year follow‐up^29^. Notwithstanding, 49% of the patients reported at least one neurological symptom, with fatigue or muscle weakness (20%) being the most frequently reported one.^29^ Anxiety or depression was reported in 26% of the patients.^29^ Similarly, a prospective multicenter cohort study showed a high frequency of self‐reported physical (74%), mental health (26%), and cognitive symptoms (16%) in COVID‐19 survivors 1 year after ICU treatment.^30^ Results of our study are in line with these findings and show similar frequencies of subjective cognitive, physical, and mental health symptoms in COVID‐19 patients 1 year after infection.

This study allows for a comparison between disease severity grades. Increased levels of fatigue and anxiety were more frequent in ICU‐admitted patients than in other patients, whereas increased levels of depression and distractibility were more common in outpatients and ICU‐admitted patients than in non‐ICU‐admitted patients. This means that not only patients with a severe initial disease course but also those with a milder course may experience subjective cognitive deficits, fatigue, and mental health symptoms in the long term. Previous studies^12^, ^31^ have discussed the necessity to screen post‐COVID‐19 patients for anxiety, depression, or post‐traumatic stress disorders, as these may relevantly influence the patients' quality of life in the long term. Findings of this study suggest that the subjective burden of COVID‐19 patients has no linear relationship with the initial disease course, and that even patients who were not hospitalized may need targeted and personalized treatment strategies, including psychological support.

Different mechanisms have been suggested to contribute to the long‐term effects of COVID‐19 on cognition. First, vascular risk factors such as cardiovascular diseases and hypertension may be involved in the development of cognitive impairments in COVID‐19 patients.^32^ Second, chronic systemic complications that can harm the brain through long‐lasting hypoxia, metabolic dysfunction, and hormonal dysregulation can contribute to post‐COVID‐19 cognitive deficits.^33^ Finally, chronic systemic inflammation has been demonstrated in COVID‐19 patients^34^ and suggested to promote cognitive decline and possibly neurodegeneration.^35^ Vascular risk factors, systemic complications, and inflammatory responses are usually increased in patients with a severe disease course. Ferritin is an inflammation marker,^36^ and hyperferritinemia in COVID‐19 patients has been associated with cognitive impairments 12 months after infection^13^ as well as with persistent pulmonary pathologies and reduced physical performance.^37^ In this study, we found in a subgroup analysis that patients with hyperferritinemia at 1‐year follow‐up perform worse than patients with normal ferritin levels in an attention task, and that hyperferritinemia in these patients is likely chronic. Despite the small sample size, these findings add to the hypothesis that long‐term cognitive performance in COVID‐19 patients might be influenced – although not exclusively – by chronic alterations of iron homeostasis.

## Limitations

This study has some limitations. First, we do not have information about pre‐existing cognitive deficits. Although very few patients presented with pre‐existing neurological disorders, we cannot exclude that some individuals already had cognitive deficits before contracting COVID‐19. This might be in particular relevant for the hospitalized patients who were older and showed a higher frequency of cognitive impairments than the outpatients. However, it should be noticed that none of the patients with pre‐existing neurological disorders was diagnosed with mild cognitive impairment or dementia. Also and more importantly, none of our patients had a history of stroke or seizures after COVID‐19, which would have affected cognition. Despite the unavailability of a neuropsychological baseline assessment prior to COVID‐19, our results clearly suggest an association between cognitive impairments and COVID‐19, although they cannot prove causality. This is supported by the fact that (1) patients' performance differ from that of age‐matched controls without COVID‐19, and that (2) the prevalence of impairments in COVID‐19 patients, defined as scores below the 10th percentile of norms in half or more of the measures used to assess a specific cognitive domain, is higher than that expected for the age‐matched general population. Secondly, patients contracted the SARS‐CoV‐2 infection between March and June 2020 and likely had the wild type or alpha variant. This reduces the generalizability of these findings to other variants. Finally, the mismatch of sample sizes across initial disease severity grades reduces the power of these findings. Due to the relatively small sample sizes, it was also not reasonable to conduct a separate regression analysis for each disease severity group on the factors that might be associated with subjective attention deficits in everyday life.

## Conclusion

This study points to a high frequency of subjective cognitive, physical, and mental health symptoms as well as of objective cognitive deficits in COVID‐19 patients 1 year after infection. In these patients, the 1‐year functional outcome was, however, good. We found that subjective cognitive deficits in everyday situations are predicted by elevated anxiety and fatigue levels more than by objective cognitive performance. In general, our findings suggest that there is no linear relationship between initial disease courses, cognitive impairments, and subjective complaints. Not all patients with cognitive deficits may be aware of their impairments in everyday life. In turn, patients with increased subjective cognitive deficits may experience above all the negative effects of anxiety and fatigue on their everyday functioning. These patients need first of all treatment of their subjective physical and mental health symptoms. Although these findings may not be generalized to recent virus variants and vaccinated patients, we advise a comprehensive neuropsychological assessment and an individualized treatment of subjective and objective cognitive symptoms to manage the possible long‐term effects of COVID‐19.

## Author contributions

Conceptualization of the study: all authors. Funding acquisition: RH. Supervision: LZ and VR. Patient samples and data collection: LZ, VR, EG, FC, PK, AD, AF, JL, PM, BH, MP, AS, KS, RB, BP, and SK. Data analysis and curation: LZ. Methodology: LZ. Visualization: LZ. Writing the original draft: LZ and VR. Review and editing of the manuscript: all authors.

## Conflict of interest

LZ reports honoraria from Novartis and Biogen as well as a research grant from EVTZ/Austrian Science Fund (FWF): IPN 135‐B, outside of the present work. VR, MD, BP, JL, PK, FC, VM, MP, AS, and EG have no conflicts of interest related to this article. AD reports honoraria from Biogen, Roche, Eisai, and Novo Nordisk, outside the submitted work. AF reports royalties from Springer Verlag, speaker fees and honoraria from Theravance Biopharma, GE Health Care, Broadview Ventures, Austrian Autonomic Society, Stopp‐HSP, Elsevier, International Parkinson Disease and Movement Disorders Society and research grants from the FWF‐Austrian Science Fund, Medical University of Innsbruck, US MSA Coalition, Dr Johannes and Hertha Tuba Foundation, and Austrian Exchange Program, outside of the present work. PM reports lecture fees from AbbVie and a grant from the Michael J Fox Foundation (MJFF), outside the submitted work. BH reports honoraria from Bial, AbbVie, and Novartis, as well as research grants from the Austrian Science Fund (FWF), outside the submitted work. KS reports personal fees from Ono Pharma UK Ltd, Teva, UCB Pharma, Lundbeck, Roche Pharma, Grünenthal, Stada, AbbVie, Ever Pharma, Licher Pharma, Biogen, and BIAL, grants and personal fees from AOP Orphan Pharmaceuticals AG and International Parkinson and Movement Disorders Society, grants from Michael J. Fox Foundation and Austrian Science Fund (FWF), outside the submitted work. RB reports a research grant from the Austrian Science Fund (FWF), outside the submitted work. SK reports supports from VASCage – Centre on Clinical Stroke Research, outside the submitted work. VASCage is a COMET Centre within the Competence Centers for Excellent Technologies (COMET) program and funded by the Federal Ministry for Climate Action, Environment, Energy, Mobility, Innovation and Technology, the Federal Ministry of Labor and Economy, and the federal states of Tyrol, Salzburg and Vienna. COMET is managed by the Austrian Research Promotion Agency (Österreichische Forschungsförderungsgesellschaft). RH reports a research grant from the Austrian Science Fund (FWF) [grant number: KLI 986].

## Supporting information


**Table S1.** Median scores and interquartile ranges (IQRs) in objective cognitive measures and in scales assessing subjective cognitive, physical, and mental health symptoms.
**Table S2.** Median scores and interquartile ranges (IQRs) in objective cognitive measures for COVID‐19 patients and age‐matched healthy controls who did not have SARS‐CoV‐2 infection.

## Data Availability

Data are available on request from the authors.
