# Supplementary material for: Objective and subjective cognitive outcomes one year after COVID‐19
Source: Ann Clin Transl Neurol. 2024 Jul 19;11(9):2360–71. doi: 10.1002/acn3.52149 (PMC11537146; doi:10.1002/acn3.52149)
Supplement: Supplementary file 1 — Table S1. Median scores and interquartile ranges (IQRs) in objective cognitive measures and in scales assessing subjective cognitive, physical, and mental health symptoms. Table S2. Median scores and interquartile ranges (IQRs) in objective cognitive measures for COVID‐19 patients and age‐matched healthy controls who did not have SARS‐CoV‐2 infection. [file ACN3-11-2360-s001.docx]

Supplementary material

Table S1. Median scores and interquartile ranges (IQRs) in objective cognitive measures and in scales assessing subjective cognitive, physical, and mental health symptoms.

|  | All  N=74 | Mild  n=24 (32%) | Moderate n=33 (45%) | Severe  n=17 (23%) |
| --- | --- | --- | --- | --- |
| MoCA | 27 (26-29) | 29 (27-29) | 27 (25-28) | 27 (25-28) |
| Attention |  |  |  |  |
| Verbal attention span, digit span forward (WMS) | 7 (6-8) | 8 (6-9) | 6 (5-8) | 7 (6-7) |
| Intrinsic alertness, median RTs in msec (TAP) | 267 (231-325) | 255 (230-298) | 277 (247-350) | 258 (223-322) |
| Phasic alertness, median RTs in msec (TAP) | 265 (235-315) | 278 (236-303) | 267 (237-331) | 246 (233-361) |
| Divided attention, omissions (TAP) | 1 (0-2) | 1 (0-2) | 1 (0-4) | 1 (0-1) |
| Executive functions |  |  |  |  |
| Verbal working memory, digit span backward (WMS) | 6 (5-7) | 6 (6-8) | 6 (5-7) | 6 (5-7) |
| Semantic verbal fluency, animals/min (RWT) | 24 (21-30) | 27 (21-34) | 23 (19-28) | 24 (21-27) |
| Phonemic verbal fluency, s-words/min (RWT) | 13 (10-16) | 14 (10-16) | 12 (9-16) | 14 (10-19) |
| Psychomotor speed, RTs in sec (TMT-A) | 26 (22-35) | 22 (16-27) | 30 (24-42) | 26 (24-35) |
| Cognitive flexibility, RTs in sec (TMT-B) | 65 (51-87) | 53 (44-65) | 71 (53-96) | 69 (54-99) |
| Memory |  |  |  |  |
| Verbal learning (NAB) | 22 (18-25) | 25 (22-28) | 20 (16-22) | 20 (17-23) |
| Verbal immediate free recall (NAB) | 7 (5-9) | 9 (8-11) | 5 (4-8) | 7 (5-8) |
| Verbal delayed free recall (NAB) | 7 (5-9) | 8 (8-11) | 5 (4-7) | 7 (5-8) |
| Verbal correct recognition (NAB) | 8 (6-11) | 10 (8-11) | 7 (4-10) | 8 (5-10) |
| Subjective cognitive, physical, and mental health symptoms |  |  |  |  |
| Subjective distractibility (FEDA-1) | 52 (43-58) | 54 (37-61) | 51 (48-58) | 50 (34-53) |
| Subjective tiredness (FEDA-2) | 35 (31-38) | 36 (32-36) | 36 (32-38) | 31 (26-36) |
| Subjective drive reduction (FEDA-3) | 25 (21-29) | 26 (20-30) | 26 (23-28) | 23 (20-28) |
| Anxiety (HADS) | 4 (2-8) | 4 (2-8) | 4 (1-8) | 5 (3-8) |
| Depression (HADS) | 2 (0-6) | 1 (0-2) | 3 (0-5) | 3 (1-8) |
| Fatigue (FAS) | 21 (18-26) | 19 (16-24) | 21 (18-26) | 24 (18-30) |

Legend: Mild = outpatients; Moderate = hospitalised patients, who were not admitted to the intensive care unit (ICU); Severe = ICU-admitted patients; MoCA = Montreal Cognitive Assessment; WMS = Wechsler Memory Scale; TAP = Tests of Attentional Performance; RWT = Regensburger Word fluency Test; TMT = Trail Making Test; NAB = Neuropsychological Assessment Battery; HADS = Hospital Anxiety and Depression Scale - German version; FEDA = Fragebogen Erlebter Defizite der Aufmerksamkeit; FAS = Fatigue Assessment Scale; RTs = reaction times.

Table S2. Median scores and interquartile ranges (IQRs) in objective cognitive measures for COVID-19 patients and age-matched healthy controls who did not have SARS-CoV-2 infection.

|  | Max. Score | COVID-19 Patients  n=74 | Healthy Controls  n=30 | P-value^b^ |
| --- | --- | --- | --- | --- |
| MoCA | 30 | 27 (26-29) | 29 (27-30) | .015 |
| Attention |  |  |  |  |
| Verbal attention span, digit span forward (WMS) | 12 | 7 (6-8) | 8 (8-9) | <.001 |
| Intrinsic alertness, median RTs in msec (TAP) | - | 267 (231-325) | 230 (217-255) | .003 |
| Phasic alertness, median RTs in msec (TAP) | - | 265 (235-315) | 244 (230-275) | .133 |
| Divided attention, omissions (TAP) | - | 1 (0-2) | 0 (0-1) | .015 |
| Executive functions |  |  |  |  |
| Verbal working memory, digit span backward (WMS) | 12 | 6 (5-7) | 7 (6-8) | .016 |
| Semantic verbal fluency, animals/min (RWT) | - | 24 (21-30) | 29 (26-34)^a^ | .008 |
| Phonemic verbal fluency, s-words/min (RWT) | - | 13 (10-16) | 20 (17-23)^a^ | <.001 |
| Psychomotor speed, RTs in sec (TMT-A) | - | 26 (22-35) | 28 (18-32)^a^ | .413 |
| Cognitive flexibility, RTs in sec (TMT-B) | - | 65 (51-87) | 63 (55-76)^a^ | .630 |
| Memory |  |  |  |  |
| Verbal learning (NAB) | 36 | 22 (18-25) | 26 (23-29) | <.001 |
| Verbal immediate free recall (NAB) | 12 | 7 (5-9) | 9 (7-11) | .002 |
| Verbal delayed free recall (NAB) | 12 | 7 (5-9) | 9 (7-10-1) | .005 |
| Verbal correct recognition (NAB) | 12 | 8 (6-11) | 11 (9-12) | <.001 |

Legend: MoCA = Montreal Cognitive Assessment; WMS = Wechsler Memory Scale; TAP = Tests of Attentional Performance; NAB = Neuropsychological Assessment Battery; RTs = reaction times; (a) = scores are available for 16 controls only; (b) = group comparisons were conducted through Mann-Whitney test.
